# Anatomic stemless shoulder arthroplasty and related outcomes: a systematic review

**DOI:** 10.1186/s12891-016-1235-0

**Published:** 2016-08-30

**Authors:** Nael Hawi, Mark Tauber, Michael Joseph Messina, Peter Habermeyer, Frank Martetschläger

**Affiliations:** 1 Department of Shoulder and Elbow Surgery, ATOS Clinic Munich, Effnerstraße 38, 81925 München, Germany; 2 Trauma Department, Hannover Medical School, Carl-Neuberg-Str. 1, 30625 Hannover, Germany; 3 Department of Traumatology and Sports Injuries, Paracelsus Medical University, Salzburg, Austria; 4 Boston Shoulder Institute/Harvard Medical School, Boston, MA USA; 5 Department of Orthopaedic Sports Medicine, Klinikum rechts der Isar, Technical University, Munich, Germany

**Keywords:** Shoulder arthroplasty, Stemless, Canal-sparing, Total shoulder arthroplasty, Shoulder arthritis, Posttraumatic shoulder arthritis

## Abstract

**Background:**

The latest generation of shoulder arthroplasty includes canal-sparing respectively stemless designs that have been developed to allow restoration of the glenohumeral center of rotation independently from the shaft, and to avoid stem-related complications. The stemless prosthesis design has also recently been introduced for use in reverse arthroplasty systems.

**Methods:**

We systematically reviewed the literature for studies of currently available canal-sparing respectively stemless shoulder arthroplasty systems. From the identified series, we recorded the indications, outcome measures, and humeral-sided complications.

**Results:**

We identified 11 studies of canal-sparing respectively stemless anatomic shoulder arthroplasty implants, published between 2010 and 2016. These studies included 929 cases, and had a mean follow-up of 26 months (range, 6 to 72 months). The rates of humeral component-related complications ranged between 0 and 7.9 %. The studies reported only a few isolated cases of complications of the humeral component. Some arthroplasty systems are associated with radiological changes, but without any clinical relevance.

**Conclusions:**

All of the published studies of canal-sparing respectively stemless shoulder arthroplasty reported promising clinical and radiological outcomes in short to midterm follow-up. Long-term studies are needed to demonstrate the long-term value of these kind of implants.

## Background

Early development of shoulder arthroplasty can be traced back to 1950s when Charles Neer, II, described the use of an implant to treat a proximal humeral fracture [1]. Eventually, the indication was widened to include osteoarthritis treatment. Since Neer’s initial prosthesis, the humeral stem design has undergone several changes. Results of total shoulder arthroplasty were first presented in 1974 [2]. The following years saw the introduction of several variations of stemmed humeral implants, which could be subdivided into four different generations. Stem design initially used a monoblock system, then changed to a modular system, followed by a shift to the use of shorter stems. Additionally, cemented fixation techniques have been replaced over time by a press-fit cementless system. The use of shorter implant stems and the elimination of humeral cement carry several advantages, including the preservation of humeral bone stock for potential revisions, performance of anatomic reconstruction regardless of posterior offset in anatomic arthroplasty, facilitating arthroplasty in cases of humeral deformity, prevention of malpositioning, and avoiding periprosthetic fractures [3].

Commonly reported stem-related complications include intraoperative humeral fracture, loosening, stress shielding, and traumatic periprosthetic fracture [4–11]. Fracture sequelae, including severe shaft-head malunion, can lead to malalignment of the shaft implantation and a failure to restore the anatomic center of rotation. In revision surgery, stem removal can present a challenge for the surgeon, potentially requiring an osteotomy or inadvertently resulting in an intraoperative fracture [5, 6, 8, 11]. Canal-sparing respectively stemless prostheses were first available in Europe in 2004. Such implants lack a conventional diaphyseal humeral stem, are based on metaphyseal fixation, and do not violate the humeral canal. In this review article, the terms “canal-sparing” respectively “stemless” refer to implant designs with metaphyseal fixation using a standard humeral neck cut, and excluding humeral head resurfacing techniques. Canal-sparing respectively stemless shoulder arthroplasty must not be confused with resurfacing techniques that aim to restore joint congruency by preserving the majority of the humeral head bone stock and implantation of a metallic cap over the remaining humeral head bone stock [3, 12–18].

Here we have systematically reviewed the current literature describing canal-sparing respectively stemless prostheses in shoulder arthroplasty, particularly with regards to clinical outcomes and complications related to the humeral components.

## Methods

The senior investigator (FM) and first author (NH) systematically scanned an online database system (Pubmed, Google Scholar) using the MeSH terms “stemless”, “shoulder replacement”, “shoulder arthroplasty”, “canal-sparing”, and “short stem”. Then the resulting list of references was reviewed to identify potential additional studies. Inclusion criteria were clinical studies including more than five patients, using cementless and stemless humeral fixation, and presenting outcomes and complications.

Statistical analysis was ineffective due to the small number of cases, as well as the use of different follow-up protocols, study designs, and outcome measures. Therefore, we performed a descriptive review, with information presented according to the different investigated prosthetic designs. For all included series, we recorded and summarized the indications, outcome measures (clinical and radiological), and complications. The scoring systems of the different studies are presented systematically. Only complications related to the humeral component are included.

## Results

Our findings are summarized in Tables 1, 2, 3, 4 and 5.

**Table 1:** Included patients and follow-up

			Implant	n = included stemless	Age (years, mean)	FU (months, mean)
2010	Huguet et al.	JSES	TESS	63	64	45.2
2013	Razmjou et al.	JSES	TESS	17	69	24
2013	Berth et Pap	JOT	TESS	41	67	30.8
2015	Maier et al.	BMC	TESS	12	68	6
2011	Kadum et al.	AOTS	TESS	22	71	14
2014	Bell et Coghlan	Int J Shoulder Surg	Mathys Affinis	38	68	12
				12	65	24
2011	Schoch et al.	Obere Extremitaet	Arthrex Eclipse	96	66	13.2
				19	62	
2012	Brunner et al.	Obere Extremitaet	Arthrex Eclipse	233	61	23.2
2015	Habermeyer et al.	JSES	Arthrex Eclipse	78	58	72
2016	Ho et al.	JSES	Simpliciti	149	66	24
2016	Churchill et al.	JBJS	Simpliciti	149	66	24

**Table 2:** Indication for stemless arthroplasty treatment

			Implant	N (stemless)	Primary osteoarthritis	Posttraumatic osteoarthritis	Osteo-necrosis	Rheumatoid arthritis	CTA	MRCT	Instability arthritis	Post infection arthtis	Arthritis due to glenoid dysplasia	Revision	
2010	Huguet et al.	JSES	TESS	63	60		3								
2013	Razmjou et al.	JSES	TESS	17	17										
2013	Berth et Pap	JOT	TESS	41	41										
2015	Maier et al.	BMC	TESS	12	12										
2011	Kadum et al.	AOTS	TESS	22	19			3							
2014	Bell et Coghlan	Int J Shoulder Surg	Mathys Affinis	50	50										
2011	Schoch et al.	Obere Extremitaet	Arthrex Eclipse	115	96	19									
2012	Brunner et al.	Obere Extremitaet	Arthrex Eclipse	233	100	70	6	16	3		29	4			5 cases couldn’t assigned
2015	Habermeyer et al.	JSES	Arthrex Eclipse	78	39	26			3		8	1	1		
2016	Ho et al.	JSES	Simpliciti	149											Not specified
2016	Churchill et al.	JBJS	Simpliciti	149	96 %	4 %

**Table 3:** Kind of stemless arthroplasty treatment and approach

			Implant	n	Approach	Hemiarthroplasty	Total shoulder arthroplasty	
2010	Huguet et al.	JSES	TESS	63	Deltopectoral	44	19	
2013	Razmjou et al.	JSES	TESS	17	Deltopectoral		17	
2013	Berth et Pap	JOT	TESS	41	Deltopectoral		41	
2015	Maier et al.	BMC	TESS	12	Deltopectoral		12	
2011	Kadum et al.	AOTS	TESS	22	Antero-Superior (Mackenzie)			Not assigned
2014	Bell et Coghlan	Int J Shoulder Surg	Mathys Affinis	50	Deltopectoral		50	
2011	Schoch et al.	Obere Extremitaet	Arthrex Eclipse	115	Deltopectoral		115	
2012	Brunner et al.	Obere Extremitaet	Arthrex Eclipse	233	Deltopectoral	114	119	
2015	Habermeyer et al.	JSES	Arthrex Eclipse	78	Deltopectoral	39	39	
2016	Ho et al.	JSES	Simpliciti	149	Deltopectoral		149	
2016	Churchill et al.	JBJS	Simpliciti	149	Deltopectoral		149

**Table 4:** Humeral implant related complication

			Implant	n	Percentage of complication	Kind of stemless **humeral** implant related complication and treatment
2010	Huguet et al.	JSES	TESS	63	7.9 %	- Five patients with a small crack of the humeral lateral cortex intraoperatively, noticed on the first postoperative radiograph, further conservative treatment
2013	Razmjou et al.	JSES	TESS	17	0 %	-
2013	Berth et Pap	JOT	TESS	41	0 %	-
2015	Maier et al.	BMC	TESS	12	0 %	-
2011	Kadum et al.	AOTS	TESS	22	0 %	-
2014	Bell et Coghlan	Int J Shoulder Surg	Mathys Affinis	50	0 %	-
2011	Schoch et al.	Obere Extremitaet	Arthrex Eclipse	115	0 %	-
2012	Brunner et al.	Obere Extremitaet	Arthrex Eclipse	233	2.3 %	- One patient with radiological and asymptomatic loosening after 24 months
2015	Habermeyer et al.	JSES	Arthrex Eclipse	78	0 %	-
2016	Ho et al.	JSES	Simpliciti	149	0 %	-
2016	Churchill et al.	JBJS	Simpliciti	149	0 %	-

**Table 5:** Outcome parameters with radiological humeral conspicuous findings (values are given in mean if not declared different)

2010	Huguet et al.	JSES	TESS	63	Constant score		Anterior active elevation (°)		ER with elbow to the side (°)		Radiological humeral component outcome
					29.6	75	96	145	20	40	Inconspicuous
2013	Razmjou et al.	JSES	TESS	17	Quick dash		WOOS		ASES		Inconspicuous
					54	23	37	85	41	82	
					Relative Constant-Murley Score		Flexion (°)		Abduction (°)		
					37	92	69	135	51	121	
					ER in neutral (°)		ER 90° abduction (°)		IR at 90° abduction (°)		
					18	54	9	61	1	33	
					Strength (lbs)						
					5	10					
2013	Berth et Pap	JOT	TESS	41	DASH score (points)		Constant score, adjusted (points)		Anteversion (°)		Inconspicuous
					62.1	47.4	40.1	73.2	81.2	115.9	
					Abduction (°)		External rotation (°)				
					68.2	105	39.1	54.4			
2015	Maier et al.	BMC	TESS	12	Constant score		Flexion (°)		Abduction (°)		-
					33.7	48	94.2	96.9	79.6	85.9	
2011	Kadum et al.	AOTS	TESS anatomic	22	Quick dash		EQ-5D		VAS for life of quality		Inconspicuous
			TESS reverse	17							
					56	34	0.36	0.73	39	66	
2014	Bell et Coghlan	Int J Shoulder Surg	Mathy Affinis	50	Constant score		DASH score		ASES score		Inconspicuous
				38	28.84	76.12	49.36	10.79	42.51	88.28	
				12	24.82	85.75	48.80	5.94	46.39	92.58	
					SPADI score		Active elevation (median) (°)				
				38	64.28	11.05	75	160			
				12	60.63	5.16	93.18	160			
2011	Schoch et al.	Obere Extremitaet	Arthrex Eclipse	115	Constant Murley score		Anteversion (°)		Abduction (°)		Inconspicuous
				96	44.7	66	118	145	82	105	
				19	34.6	57	110	140	75	100	
					External rotation (°)						
				96	15	41					
				19	15	38					
2012	Brunner et al.	Obere Extremitaet	Arthrex Eclipse	233	Constant Murley score (%)		Flexion (°)		Abduction (°)		7.2 % of cases radiolucency between the head and the screw without clinical consequences
					51.6	78.9	105	128	80	120	
					External rotation (°)						
					22	37					
2015	Habermeyer et al.	JSES	Arthrex Eclipse	78	Constant Murley score (%)		Flexion (°)		Abduction (°)		Incomplete radiolucent line of the humeral component smaller than 2 mm in one patient, in three patients partial osteolyses under the superior part of the humeral component without loosening, decreased density of cancellous bone in the greater tuberosity with the AP view in 34.9 % without influence on shoulder function.
					38.1	75.3	114	141	74	130	
					External rotation (°)						
					25	44					
2016	Ho et al.	JSES	Simpliciti	149	Constant Murley score (adjusted)		Constant Murley score (adjusted)		Constant Murley score (adjusted)		Inconspicuous
					<60 y		60–69 y		>70 y		
					51.2	90.5	55.9	105.6	58.2	110.6	
					ASES score		ASES score		ASES score		
					<60 y		60–69 y		>70 y		
					33.2	84.1	39.6	94.3	39.1	93.1	
					Simple shoulder test		Simple shoulder test		Simple shoulder test		
					<60 y		60–69 y		>70 y		
					4	10.1	4.3	11	4.6	10.7	
					External rotation (°)		External rotation (°)		External rotation (°)		
					<60 y		60–69 y		>70 y		
					35.4	54.9	28.8	55.8	31.3	58.2	
					Scapular plane (°)		Scapular plane (°)		Scapular plane (°)		
					<60 y		60–69 y		>70 y		
					107.1	142.4	102.3	149.6	100.9	144.5	
2016	Churchill et al.	JBJS	Simpliciti	149	Constant Murley Score (adjusted, %)		ASES score		Simple shoulder Test		Inconspicuous
					55.6	104.1	38.2	91.9	4.3	10.8	
					Pain VAS Score		Scapular plane (°)		Eternal rotation (°)		
					5.9	0.5	102.8	146.6	30.9	56.4	

## Biomet Total Evolutive Shoulder System

**Fig. 1:**
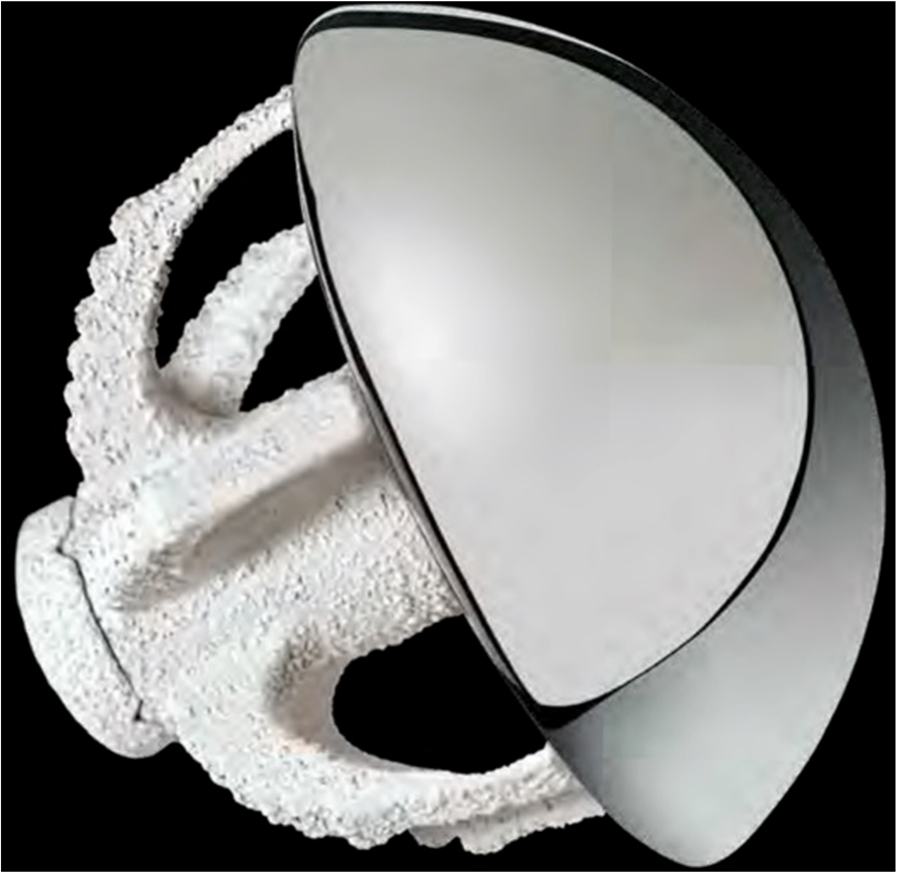
The Biomet Total Evolutive Shoulder System (TESS; Biomet, Warsaw, IN, USA) (Figure provided by the manufacturer)

The first available canal-sparing respectively stemless implant was the Biomet Total Evolutive Shoulder System (TESS, Biomet, Warsaw, IN, USA), which was first used in Europe in 2004 (Fig. 1). The TESS is a three-component system, which includes an impaction-implanted 6-armed corolla that is porous to improve bone ingrowth. We identified five studies using the TESS, which were published between 2010 and 2016, and included a total of 155 patients. Follow-up times ranged from 6 to 45 months. Table 2 presents the surgical indications. All studies showed clinical improvement after arthroplasty compared to the preoperative status.

In 2010, Huguet et al. [19] first reported on 63 cases with a minimum follow-up of three years. Concerning the TESS stemless humeral implant, the authors report that in five cases the lateral cortex cracked during surgery without any sign of instability, and that each of these cases healed without further intervention. No other humeral implant-associated complications or problems were noted.

The remaining four identified studies reported no complications related to the TESS humeral implant. Kadum et al. [20] analyzed 56 patients, among whom 22 received an anatomic TESS prosthesis, with a mean follow-up of 14 months. Razmjou et al. [21] compared the anatomic TESS prosthesis (*n* = 17) to the Bigliani-Flatow (*n* = 40) and the Neer II prosthesis (*n* = 22), showing no significant differences in outcome between groups, with a mean follow-up of 24 months. Berth and Pap [22] compared the anatomic TESS prosthesis to the Mathys Affinis stemmed prosthesis, with 41 patients in each group and a mean follow-up of 30 months. Their results revealed no statistically significant differences in outcome. Finally, Meier et al. [23] compared the anatomic TESS prosthesis to the anatomic stemmed Aequalis Shoulder prosthesis (Tournier, Lyon, France), with 12 cases per group and a 6-month follow-up. They reported comparable results for both groups based on the Constant Score.

## Mathys Affinis Short Stemless Prosthesis

**Fig. 2:**
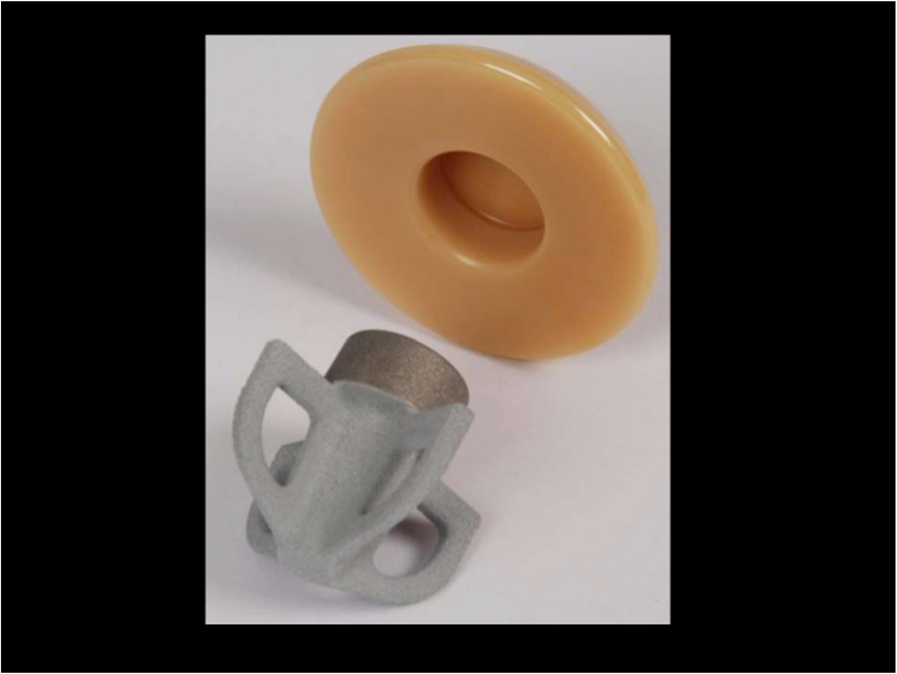
The Mathys Affinis Short Stemless Prosthesis (Mathys, Betlach, Switzerland) (Figure provided by the manufacturer)

The Mathys Affinis Short Stemless Prosthesis (Mathys, Betlach, Switzerland) was first available on the European market in 2009 (Fig. 2). This arthroplasty system has two components: a humeral metaphyseal implant and a ceramic humeral head. The metaphyseal implant comprises four wings composed of a rough porous titanium structure, with an osteoconductive calcium phosphate coating to improve bone ingrowth. This part is inserted with impaction.

In the only published study using this implant, Bell and Coghlan [24] investigated 50 cases with an indication of primary osteoarthritis. They reported a 24-month follow-up for 12 cases, and a 12-month follow-up for 38 cases. No prosthesis-related intraoperative or postoperative complications were reported.

## Arthrex Eclipse Prosthesis

**Fig. 3:**
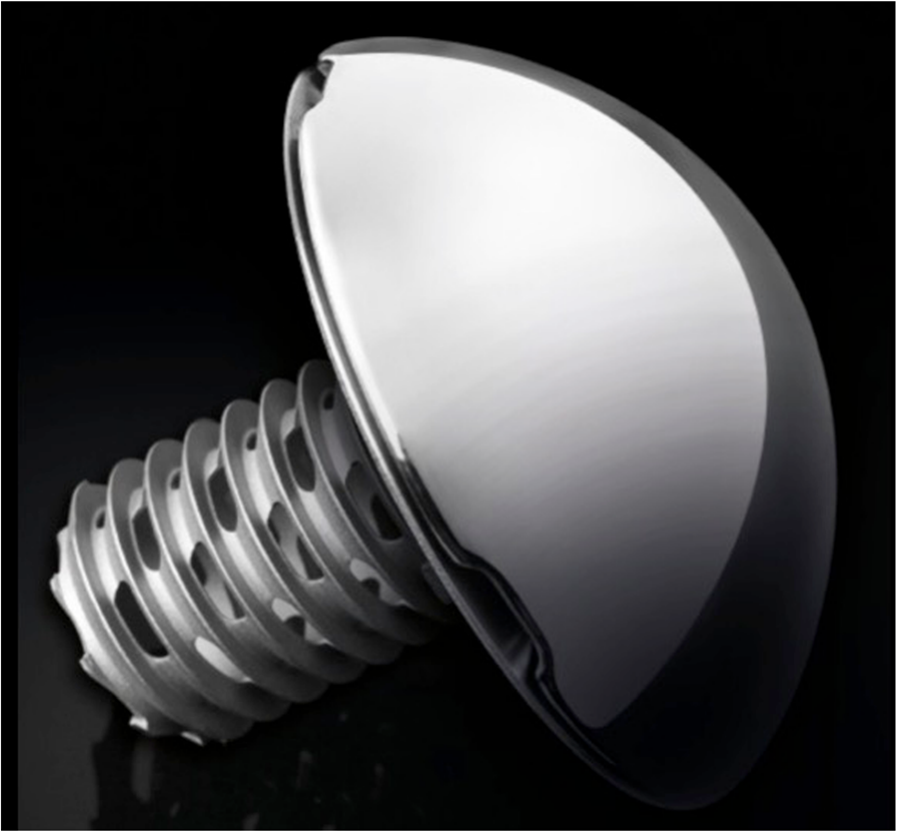
The Arthrex Eclipse Prosthesis (Arthrex, Naples, USA) (Figure provided by the manufacturer)

The Arthrex Eclipse Prosthesis (Arthrex, Naples, USA) was introduced in 2005 (Fig. 3). This prosthesis comprises three components. A fully threaded, cylindrical central cage unit is inserted over a collar-bearing baseplate (trunnion) for metaphyseal fixation. The trunnion covers the resection plane at the anatomical neck, and joins cortical support. The third component is a corresponding humeral head. In contrast to the other described implants, the Arthrex Eclipse is the only available stemless system that offers screw-in insertion.

Between 2011 and 2015, three studies investigated the Eclipse prosthesis, including a total of 426 procedures with a mean follow-up ranging from 13 to 72 months. Table 2 presents the specific surgical indications. Schoch et al. [25] analyzed 115 cases, and reported that all of the radiographs evaluated at the 12-month follow-up were inconspicuous with regards to loosening or radiolucent lines. Brunner et al. [26] published their experience of using the Eclipse prosthesis in 233 patients after a mean follow-up of 23 months. They describe one case in which the implant loosened after 24 months, and they noted radiolucency between the head and the screw in 7.2 % of cases. However, these radiological changes did not appear to have any clinical relevance.

In the most recent study of the Eclipse prosthesis, Habermeyer et al. [27] analyzed 78 patients with a minimum follow-up of 5 years. They reported that one patient showed an incomplete radiolucent line of the humeral component of smaller than 2 mm. Additionally, three patients exhibited partial osteolysis under the superior part of the humeral component, without loosening of the component. Among the patients, 34.9 % showed changed cancellous bone density in terms of stress shielding at the greater tuberosity on the AP view, without clinical significance. No implant-specific complications were observed related to the Eclipse prosthesis.

## Simpliciti Stemless Prosthesis

**Fig. 4:**
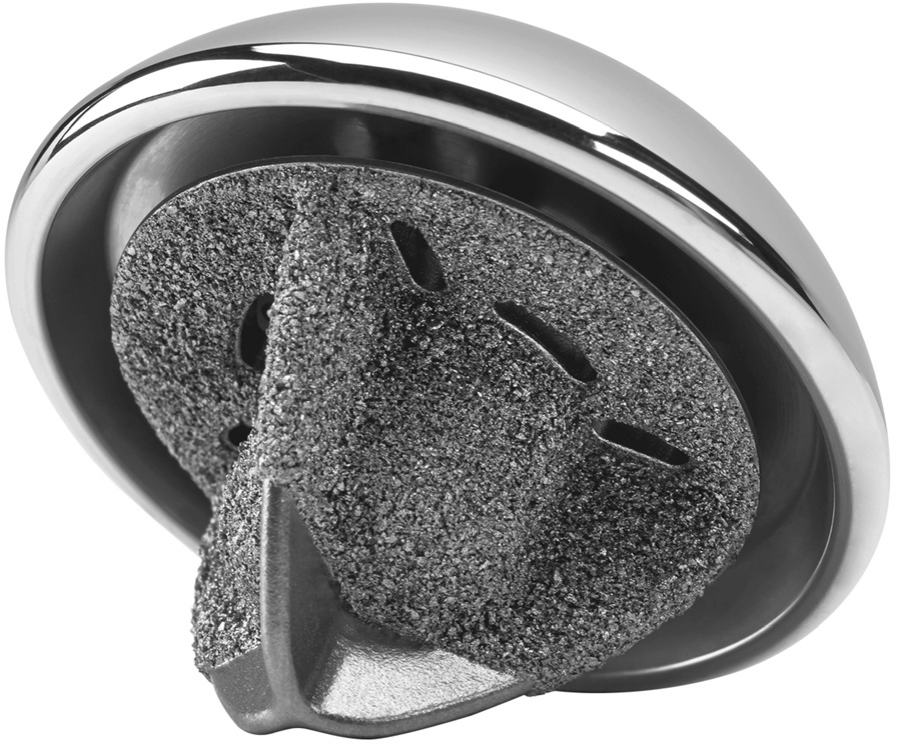
The Simpliciti Stemless Prosthesis (Tornier, Bloomington, MN, US) (Figure provided by the manufacturer)

Clinical use of the Simpliciti Stemless Prosthesis (Tornier, Bloomington, MN, US) began in France in 2010 (Fig. 4). It is presently the only FDA-approved stemless respectively canal-sparing implant. It comprises two pieces—a humeral implant and a head implant—which are available in different sizes. Churchill et al. [27] and Ho et al. [18, 28] both recently published results of canal-sparing respectively stemless shoulder arthroplasty with a 24-month follow-up in a total of 298 patients. Table 2 summarizes the indications. Both studies demonstrated improved outcomes, and no humeral-sided complications.

## Discussion

Six different canal-sparing respectively stemless humeral implants are presently available on the market, four of which are described in published studies. The canal-sparing respectively stemless shoulder prosthesis with metaphyseal anchoring is a relatively new concept that reportedly provides good outcomes that are comparable to stemmed designs in short and midterm evaluations [19, 21–23, 27]. All of the presently reviewed studies demonstrated substantial improvement after replacement. Moreover, the studies that compared the canal-sparing respectively stemless design to stemmed implants showed no differences in outcomes related to the humeral component [21–23].

The indications for canal-sparing respectively stemless prosthesis are the same as for stemmed systems. Canal-sparing respectively stemless implants cannot be used in cases with poor bone quality, metaphyseal cysts, osteopenia, osteoporosis, or other metabolic bone diseases [29], or in cases with fractures in the metaphyseal area that disturb adequate bony in-growth or primary implant stability. However, there is not yet any test available to objectively determine bone quality pre- or intraoperatively [29]. Churchill et al. [18] described the use of a “thumb test” in which bone quality is intraoperatively assessed by compressing the surface of the neck cut with the thumb. Overall, the presently reviewed studies reported only a few isolated cases of loosening of the stemless component.

Among the various investigated canal-sparing respectively stemless prostheses, the main difference in design is that the Eclipse is inserted over a screw, while the TESS, the Simpliciti, and the Affinis prostheses are inserted using an impaction technique. It appears that the mechanism of force transmission when the Eclipse prosthesis is inserted over a screw differs from that during impaction implantation of the corolla of the TESS, the wings of the Affinis, or the Simpliciti prosthesis. This difference may explain the observed differences in the surrounding humeral bone, with changed bone mineral density seen on radiographs, which could be interpreted as stress shielding beneath the trunnion with the Eclipse prosthesis. These findings are not correlated with any negative clinical symptoms, and seem to be purely a radiographic phenomenon, at least at the midterm follow-up. Subgroup analysis of these patients reported by Habermeyer et al. [27] revealed no statistical significance in the patient cohort, with a minimum follow-up of 5 years.

One major advantage of the canal-sparing respectively stemless prosthesis design is that it can potentially be used in post-traumatic and deformity cases regardless of the humeral head–shaft configuration. Restoration of the glenohumeral center of rotation independently from the shaft is a key goal in secondary shoulder arthroplasty for fracture sequelae treatment [30]. Malunion resulting in metaphyseal–diaphyseal malalignment can make it difficult or even impossible to implant a stemmed or even a short-stemmed prosthesis. In such cases, corrective osteotomies are associated with poor results [31]. Reports of these types of special cases are limited to only rare single cases within the studies. Other advantages of the canal-sparing respectively stemless prosthesis are that it can preserve bone stock of the proximal humerus, as well as avoid humeral stem-related complications in revision cases requiring stem removal. In a commentary, Athwal summarized the advantages of the canal-sparing respectively stemless implant as a theoretically decreased surgical time, less blood loss, bone preservation, and lower risk of intraoperative and potentially postoperative periprosthetic fractures. Canal-sparing respectively stemless prostheses are also suitable for posttraumatic joint reconstruction and, when needed, explantation is easier compared to with the stemmed version. Following explantation, a stemless prosthesis can be replaced by a standard-length primary implant [29].

For the purpose of this review, we chose to include all available literature evaluating anatomic canal-sparing respectively stemless humeral components in shoulder arthroplasty. This review article refers to only implant designs with metaphyseal fixation, and excludes humeral head resurfacing. It is much easier to achieve glenoid exposure for glenoid component implantation using canal-sparing respectively stemless implants compared to with humeral head resurfacing [3, 20].

It must be noted that, all reviewed studies provide only short or midterm results, and include only a limited number of patients. Additionally, some studies were reported by the designer or co-developer of the investigated implant, which suggests the possibility of a certain bias. There are presently ongoing IDE trials, which will provide more robust and high-quality data on this topic.

## Conclusions

All of the published studies describing anatomic canal-sparing respectively stemless shoulder replacement showed promising clinical and radiological outcomes over short to midterm follow-up periods. To date, the available literature lacks well-designed clinical studies with at least midterm results.

## Abbreviations

ER: external rotation
IR: internal rotation
CTA: cuff tear arthropathy
MRCT: massive rotator cuff tear
VAS: Visual Analog Scale
SPADI Index: Shoulder Pain and Disability Index
DASH Score: Disabilities of the Arm, Shoulder and Hand Score
ASES Score: American Shoulder and Elbow Surgeons Shoulder Score
WOOS Index: Western Ontario Osteoarthritis Shoulder Index
e.g.: For example
etc.: Et cetera
